# Calcium channel blocker-associated pulmonary toxicity: epidemiology, mechanisms, and clinical management

**DOI:** 10.3389/fphar.2026.1892392

**Published:** 2026-09-21

**Authors:** Qiu Jiang, Wen-Hui Cui, Jia-Yi He, Zhi-Qi Xie, Jun-Jie Xu, Xin-Yue Fu, Xiao-Qin Wang

**Affiliations:** 1 Department of Pharmacy, The Quzhou Affiliated Hospital of Wenzhou Medical University, Quzhou People’s Hospital, Quzhou, China; 2 Pharmacy Department, Sinopharm Tongmei General Hospital, Datong, China; 3 Department of Pharmacy, The First People’s Hospital of Wuyi County, Jinhua, China; 4 School of Medicine, Hangzhou City University, Hangzhou, China; 5 Department of Pharmacy, The Second Affiliated Hospital of Jiaxing University, Jiaxing, China

**Keywords:** acute respiratory distress syndrome, calcium channel blockers, drug overdose, pharmacovigilance, pulmonary toxicity

## Abstract

Calcium channel blockers are first-line medications for the treatment of hypertension, angina pectoris, and arrhythmias, and are generally well-tolerated. However, in recent years, there have been reports indicating that CCBs may cause severe pulmonary toxicity, such as non-cardiogenic pulmonary edema and acute respiratory distress syndrome. This article employs a comprehensive review methodology to systematically summarize the clinical types, incidence, risk factors, mechanisms of injury, and management strategies for CCB-related pulmonary toxicity. Current evidence indicates that pulmonary toxicity is extremely rare at standard therapeutic doses; however, the incidence increases significantly in cases of acute overdose and among specific populations (pregnant women, patients with underlying cardiopulmonary diseases). Pharmacovigilance studies have identified significant signals suggesting an association between nifedipine, diltiazem, and verapamil to pulmonary edema, with amlodipine having the highest number of related clinical reports. Major risk factors include drug overdose, pregnancy, underlying cardiopulmonary disease, and differences among CCB subclasses. Toxicity associated with dihydropyridine CCBs is primarily characterized by vasodilatory shock, whereas non-dihydropyridine CCBs exhibit a combination of cardiac depressant and vasodilatory effects. Mechanisms involve hemodynamic changes, increased capillary permeability, inflammatory activation, and alveolar epithelial dysfunction. Early recognition, drug discontinuation, selection of vasoactive agents based on the type of shock, and life support are key to improving prognosis.

## 1 Introduction

Calcium channel blockers (CCBs) selectively block L-type calcium channels, thereby inhibiting the influx of calcium ions into vascular smooth muscle and cardiac muscle cells. This produces vasodilation, negative inotropic effects, negative chronotropic effects, and atrioventricular block, making them first-line medications for treating conditions such as hypertension, angina pectoris, and arrhythmias (Carey et al., 2022; Joshi and de Lemos, 2021; Font et al., 2026; Peng and Zei, 2024). Based on their chemical structure, CCBs are primarily classified into dihydropyridines (DHPs) (e.g., nifedipine, amlodipine, and felodipine) and non-dihydropyridines (non-DHPs) (e.g., verapamil and diltiazem), which exhibit significant differences in vascular and cardiac selectivity (Font et al., 2026; Frishman, 2007). L-type calcium channels (CaV1.1-CaV1.4) are a class of voltage-gated ion channels characterized by high-voltage activation and sustained inward calcium currents. The pore-forming α1 subunit, encoded by the CACNA1S, CACNA1C, CACNA1D, and CACNA1F genes, confers unique biophysical properties and pharmacological sensitivities to these channels. Although the CaV1.2 and CaV1.3 subtypes are primarily expressed in cardiac myocytes and vascular smooth muscle cells, their distribution within these tissues differs significantly, which forms the basis for the varying tissue selectivity of CCBs. DHPs have a higher affinity for the inactive state of the channels, which is more prevalent in vascular smooth muscle; therefore, they produce a potent vasodilatory effect while having minimal direct effects on the heart. In contrast, non-DHPs drugs exhibit greater selectivity for the open state of the channels in myocardial and sinoatrial node tissues, which explains their significant negative effects on heart rate, conduction, and contractility. By reducing intracellular calcium availability, CCBs attenuate excitation-contraction coupling in both vascular smooth muscle and cardiac myocytes. The differential tissue distribution of L-type calcium channels, predominantly in vascular smooth muscle for DHPs and in both cardiac and vascular tissues for non-DHPs, underlies their distinct pharmacological and toxicological profiles (Font et al., 2026; Frishman, 2007). Specifically, DHPs exert potent vasodilatory effects with minimal cardiac depression, whereas non-DHPs produce balanced cardiac and vascular effects, including negative chronotropic and inotropic actions. DHPs are available only in oral formulations, whereas non-DHPs are available in both intravenous (IV) and oral (PO) formulations; IV administration is typically reserved for acute indications such as arrhythmias or hypertensive emergencies.

Although CCBs are generally well-tolerated, their potential for pulmonary toxicity is increasingly drawing clinical attention. Adverse drug reactions are critical factors affecting patient prognosis and treatment adherence; their early identification and effective management are essential for enhancing medication safety in clinical practice. Since the 1980s, there have been ongoing case reports describing severe events such as acute dyspnea and hypoxemia following CCB therapy, ultimately diagnosed as non-cardiogenic pulmonary edema (NCPE) or even acute respiratory distress syndrome (ARDS) (Chaouat et al., 1996; Leesar et al., 1994; Siddiqi et al., 2014; Lindeman et al., 2020; Prigogine et al., 1991; Gillmer and Kark, 1980; Sridhar et al., 2025). Drug-induced pulmonary edema (DIPE) can only be diagnosed after ruling out other causes such as shock, aspiration, or severe pneumonia (Zhong et al., 2025). Although these events are rare, they can be life-threatening, and their clinical manifestations resemble those of heart failure or pulmonary infection, making misdiagnosis likely.

The importance of identifying and managing CCB-related pulmonary toxicity lies in (Carey et al., 2022): timely differentiation to avoid inappropriate treatment (Joshi and de Lemos, 2021); the condition may progress rapidly, requiring intensive care support (St-Onge et al., 2017; Simpson and Cole, 2024; Pandit et al., 2025); and (Font et al., 2026) identifying risk factors aids in prevention. In recent years, pharmacovigilance studies utilizing databases such as the FDA Adverse Event Reporting System (FAERS) have provided new real-world evidence regarding CCB-related pulmonary toxicity (Zhong et al., 2025; Ju et al., 2025). This review aims to systematically summarize the clinical characteristics, incidence, risk factors, potential mechanisms, and management strategies of CCB-related pulmonary toxicity to provide guidance for the safe clinical use of these medications.

## 2 Literature search strategy

This review takes the form of a comprehensive narrative review rather than a formal systematic review or scoping review. In May 2026, we conducted a systematic literature search using the PubMed database. For a complete search strategy (including Boolean operators (AND/OR), MeSH terms, free-text terms, and database-specific syntax), see Supplementary Table S1. The search scope covered literature published between 1980 and 2026, with a particular focus on evidence published between 2020 and 2026 to include both pioneering case reports and the related pharmacovigilance data. Inclusion Criteria (Carey et al., 2022): Original studies (case reports, case series, cohort studies, case-control studies, pharmacovigilance studies) reporting CCB-related pulmonary adverse reactions (Joshi and de Lemos, 2021); Experimental studies investigating the mechanisms of CCB-induced pulmonary toxicity (Font et al., 2026); Articles published in peer-reviewed journals in English or Chinese. Exclusion Criteria (Carey et al., 2022): Reviews, editorials, conference abstracts, and animal studies (Joshi and de Lemos, 2021); Pulmonary edema clearly caused by cardiac factors (e.g., acute myocardial infarction, decompensated heart failure) (Font et al., 2026); Literature for which the full text was unavailable. In addition, the reference lists of the included articles were manually screened. The total number of studies ultimately included in this review was 32 (Figure 1).

**FIGURE 1:**
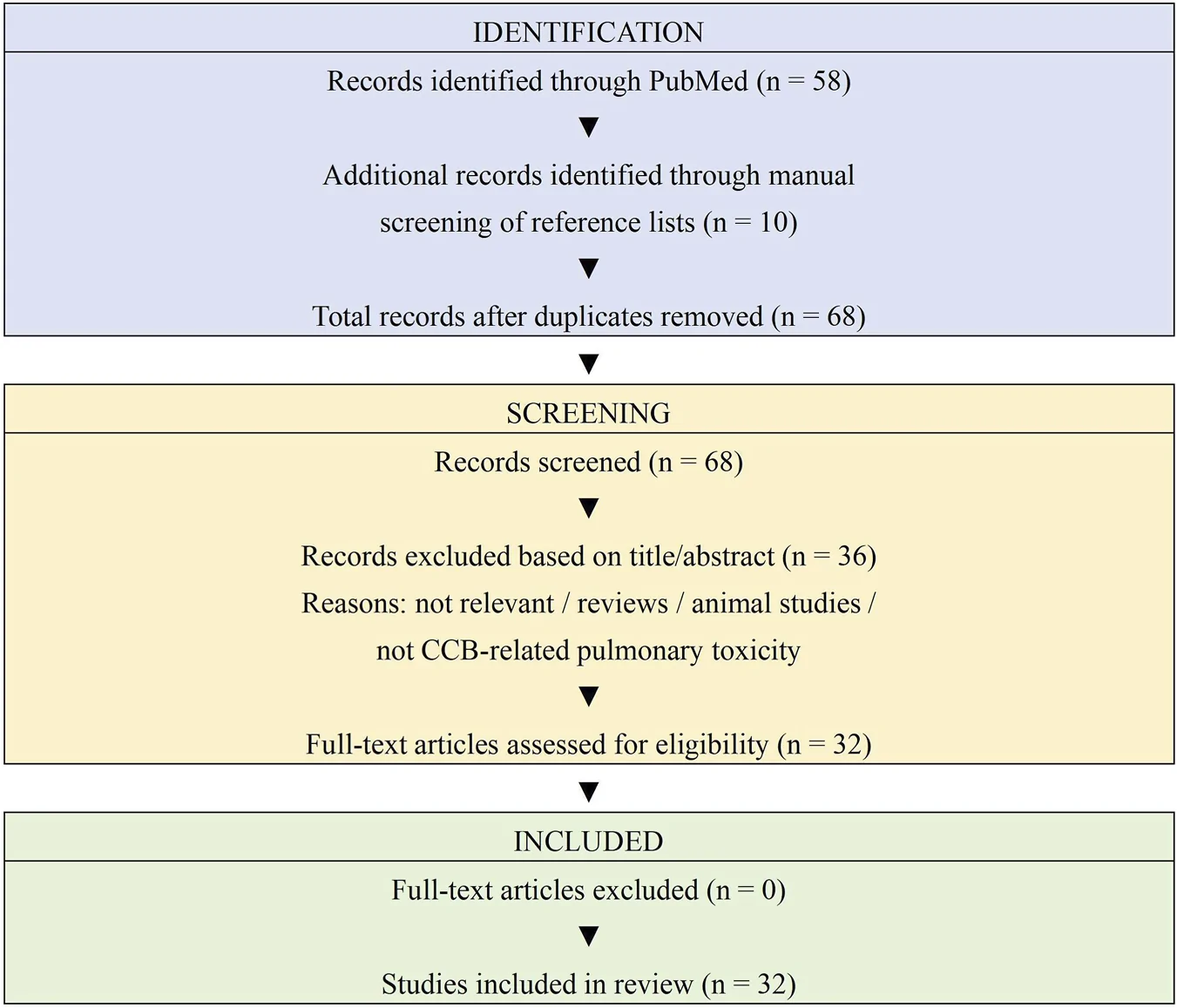
PRISMA-style flowchart of the literature screening and selection process.

## 3 Results

### 3.1 Types of injury

CCB-related pulmonary toxicity primarily comprises two types: NCPE and ARDS. NCPE is characterized by abnormal fluid accumulation in the alveoli and interstitium in the absence of evidence of left ventricular dysfunction; its underlying cause is damage to the alveolar-capillary barrier, which distinguishes it from cardiogenic pulmonary edema caused by heart failure (Ware and Matthay, 2000; Malek and Soufi, 2023). Multiple case reports have described NCPE associated with excessive CCB intake, involving nifedipine (up to 900 mg) (Chaouat et al., 1996; Prigogine et al., 1991; Gillmer and Kark, 1980; Herrington et al., 1986), amlodipine (5–500 mg, with some cases involving standard therapeutic doses) (Lindeman et al., 2020; Zambetti and Parrish, 2025; Abboud et al., 2012; Bringgold and Long, 2022), verapamil (oral 2,400–8,640 mg) (Leesar et al., 1994; Siddiqi et al., 2014; Martin et al., 2014; Howarth et al., 1994; Brass et al., 1996; Sami Karti et al., 2002), and diltiazem (Humbert et al., 1991). Amlodipine can also induce severe NCPE at standard therapeutic doses (5 mg/day), suggesting that individual susceptibility may play a significant role (Abboud et al., 2012).

ARDS is a more severe form of inflammatory lung injury characterized by bilateral diffuse pulmonary infiltrates and hypoxemia (PaO_2_/FiO_2_ ≤ 300) (Ranieri et al., 2012). ARDS may develop following CCB overdose, particularly in cases of verapamil poisoning, which has been clearly documented (Martin et al., 2014). ARDS can lead to pulmonary inflammation and fibrosis and, in severe cases, may be life-threatening.

### 3.2 Latency period and incidence

The latency period for CCB-induced pulmonary toxicity varies widely among cases, ranging from half an hour to several days (Herrington et al., 1986; Zambetti and Parrish, 2025), and detailed records are lacking in some cases. This suggests that the occurrence of pulmonary toxicity may be associated with multiple factors, including drug dosage and patient-specific variations. Based on existing case reports, it is not possible to clearly distinguish the onset times between DHP and non-DHP CCBs; this may be due to differences in dosage, formulation (immediate-release versus extended-release), and individual patient factors. non-DHP CCBs (verapamil, diltiazem) can be administered intravenously or orally, whereas DHP drugs are available only in oral formulations.

At standard therapeutic doses, the reported occurrence of CCB-related pulmonary toxicity is extremely low. However, the frequency increases significantly under specific circumstances, such as acute overdose and pregnancy. Regarding acute overdose, a cohort study by Lindeman et al. (2020) of 19 patients with severe amlodipine poisoning showed a reported proportion of NCPE of 47% (9/19) (Lindeman et al., 2020). Toxic concentrations (plasma amlodipine >0.25 mg/L) are far higher than the therapeutic concentration range (0.001–0.024 mg/L). In pregnant women, Spiesser-Robelet et al. found that among 213 pregnant women receiving nifedipine for tocolysis, the reported occurrence of severe cardiopulmonary adverse events was 8.9%, with 6 cases (2.8% of the total cohort) representing definite acute pulmonary edema (Spiesser-Robelet et al., 2015).

### 3.3 Pharmacovigilance research evidence

Zhong et al. analyzed FAERS data from 2004 to 2024 and identified 37 drugs with a risk of DIPE among 173 target drugs. Among these, nifedipine ranked third in the reported odds ratio (ROR) ranking, and diltiazem was also identified, with a median onset time of less than 30 days. Although amlodipine did not reach the positive signal threshold in the disproportionate analysis, it ranked second in DIPE reporting frequency with 456 reports; this may partly reflect the current high clinical usage of amlodipine, but it also suggests that DIPE-related pulmonary edema events warrant clinical attention. Furthermore, the study noted that verapamil overdose can promote the development of pulmonary edema by causing peripheral vasodilation and fluid redistribution through its effects on vascular smooth muscle (Zhong et al., 2025). Ju et al. conducted a disproportionate analysis specifically for nifedipine, revealing a very strong signal for acute pulmonary edema in pregnant women (ROR = 67.23) (Ju et al., 2025).

### 3.4 Primary drugs of concern

Based on a comprehensive review of pharmacovigilance signals, cohort studies, and case reports, the following four CCBs are most commonly associated with pulmonary toxicity. Nifedipine is most commonly associated with pulmonary toxicity in cases of acute overdose (Herrington et al., 1986), potential pulmonary arterial hypertension (Chaouat et al., 1996; Prigogine et al., 1991), and use as a tocolytic agent in pregnant women (Gillmer and Kark, 1980). FAERS analysis indicates that it ranks third in DIPE signal strength (Zhong et al., 2025), and the signal for acute pulmonary edema is particularly strong in pregnant women (ROR = 67.23) (Ju et al., 2025). However, pregnancy itself is a dynamic state characterized by fluid shifts, hormonal changes, and alterations in cardiovascular physiology, factors that may increase the risk of DIPE. In obstetric clinical practice, nifedipine should only be considered a causative factor for DIPE after other common pregnancy-related causes, including pre-eclampsia, peripartum cardiomyopathy, and iatrogenic fluid overload, have been ruled out. Amlodipine is currently the CCB with the highest number of reported cases of pulmonary toxicity in clinical reports (Zhong et al., 2025). The reported proportion of NCPE can reach 47% in cases of severe poisoning (Lindeman et al., 2020). Case reports describe delayed onset (3 days after ingestion), severe NCPE induced by low doses (5 mg/day) with recurrence upon re-exposure (Abboud et al., 2012), and cases complicated by diffuse alveolar hemorrhage (Bringgold and Long, 2022).

Overdose of verapamil can lead to NCPE or ARDS (7, 24). FAERS has identified signals associated with the risk of DIPE, and clinical observations suggest that the mechanism may be related to vasodilation and fluid redistribution (Zhong et al., 2025). For diltiazem, there have been case reports of NCPE following overdose (Humbert et al., 1991), and FAERS identified a median time to onset of less than 30 days (Zhong et al., 2025). Furthermore, there have been case reports of nicardipine inducing pulmonary edema when used as a tocolytic agent (Bal et al., 2004); fatal adverse events associated with felodipine overdose have also been documented in the literature (Lota et al., 2008).

### 3.5 Risk factors

Based on an analysis of existing case reports and pharmacovigilance data, the primary risk factors for CCB-associated pulmonary toxicity include drug dose and formulation, underlying medical conditions, specific physiological states, differences among CCB subclasses and drug interactions. Among these, acute overdose is the most well-established risk factor (Lindeman et al., 2020; Isbister et al., 2025). Extended-release formulations may prolong the duration of toxicity. It is worth noting that even low doses (5 mg/day) of amlodipine may induce severe NCPE, suggesting the presence of individual susceptibility (Abboud et al., 2012).

Underlying conditions such as pulmonary arterial hypertension (Chaouat et al., 1996; Prigogine et al., 1991), hypertrophic obstructive cardiomyopathy (Natarajan et al., 1990), aortic stenosis, and severe cardiac structural or functional abnormalities can all increase the risk of pulmonary edema (Gillmer and Kark, 1980). Pregnancy, particularly when nifedipine is used to inhibit uterine contractions, is another important risk factor (Gillmer and Kark, 1980; Spiesser-Robelet et al., 2015). Physiological changes during pregnancy, including increased plasma volume, decreased colloid osmotic pressure, and elevated cardiac output, may lower the threshold for the development of pulmonary edema; therefore, this factor should be taken into account when evaluating the role of nifedipine as a precipitating factor. FAERS data indicate a very strong signal for acute pulmonary edema in pregnant women (ROR = 67.23) (Ju et al., 2025). non-DHP CCBs (diltiazem and verapamil) are generally more toxic and may carry a higher risk of pulmonary toxicity (Isbister et al., 2025). Concomitant use of other negative inotropic agents or CYP3A4 inhibitors may also increase the risk of toxicity (Isbister et al., 2025).

### 3.6 Mechanisms of injury

Pulmonary edema results from an imbalance between the fluid that infiltrates the alveolar interstitium and the fluid that is eliminated via lymphatic drainage and alveolar fluid clearance. The alveolar-capillary barrier, composed of microvascular endothelium and alveolar epithelium, maintains strict fluid homeostasis under physiological conditions. Impairment of this barrier, whether due to increased hydrostatic pressure, increased capillary permeability, or impaired epithelial fluid transport, leads to the accumulation of protein-rich fluid in the alveolar space. L-type voltage-sensitive calcium channels are distributed at key locations and can influence multiple aspects of this barrier’s function. Their blockade can increase pulmonary capillary hydrostatic pressure through systemic vasodilation and fluid redistribution; their effects on endothelial calcium signaling may regulate endothelial permeability; and their action on alveolar epithelial cells may impair active sodium transport and fluid clearance. Furthermore, the hemodynamic consequences of CCB-induced vasodilation, particularly in cases of acute overdose, may lead to a rapid increase in pulmonary blood volume and pulmonary artery pressure, thereby overwhelming the lung’s compensatory mechanisms and promoting the development of edema. These pathways are also influenced by redox-sensitive signaling mechanisms, as studies have shown that oxidative stress can modulate the activity of protein kinases A and G, which in turn regulate vascular tone and endothelial barrier function (Burgoyne and Eaton, 2010). Taken together, these interrelated mechanisms provide a theoretical framework for understanding how L-type calcium channel blockade leads to the development of pulmonary edema in various clinical contexts.

The mechanisms underlying CCB-induced pulmonary toxicity are multifactorial, with different subclasses emphasizing different factors (Frishman, 2007; Leesar et al., 1994; Ware and Matthay, 2000; Isbister et al., 2025). Given the significant differences in tissue selectivity between DHP and non-DHP CCBs, the pathophysiological mechanisms are best discussed separately for each subclass. For DHP CCBs (e.g., nifedipine, amlodipine), the predominant mechanism is excessive peripheral vasodilation leading to distributive shock and reflex tachycardia (Isbister et al., 2025). These agents have greater selectivity for vascular L-type calcium channels, resulting in potent vasodilatory effects with relatively less direct cardiac depression. The consequent reduction in systemic vascular resistance and effective arterial blood volume triggers a compensatory increase in cardiac output, which, in the presence of increased pulmonary capillary permeability, may promote fluid extravasation into the alveolar space. Some studies have suggested that DHPs may increase microvascular permeability through enhanced nitric oxide release from vascular endothelium, though this mechanism requires further validation (Isbister et al., 2025).

For non-DHP CCBs (e.g., verapamil, diltiazem), the toxicity profile involves a combination of cardiac depression and vasodilation, reflecting their relatively balanced affinity for L-type calcium channels in blood vessels and the heart (Isbister et al., 2025). These drugs exert negative inotropic, negative chronotropic, and atrioventricular conduction-delaying effects, which may lead to bradycardia, hypotension, and reduced cardiac output. The combination of impaired cardiac function and peripheral vasodilation creates a hemodynamic environment that promotes pulmonary congestion and edema.

Shared mechanisms across both subclasses include increased capillary permeability, inflammatory activation, and alveolar epithelial dysfunction. A key question is whether, given that elevated intracellular calcium concentrations are widely recognized as a trigger for barrier disruption, CCBs, which block calcium influx, actually increase endothelial permeability. The answer lies in the cell-type-specific and channel-specific nature of calcium signaling in pulmonary microvessels. In pulmonary endothelial cells, Ca^2+^ entering through TRPV4 channels (rather than L-type voltage-gated calcium channels) is the primary driver of increased permeability and alveolar fluid accumulation. Activation of TRPV4 triggers a localized Ca^2+^ signal that disrupts the endothelial barrier through cytoskeletal reorganization and disassembly of focal adhesions; these effects are independent of L-type channel activity. In alveolar epithelial cells, calcium overload impairs active sodium transport and surfactant production, further compromising fluid clearance capacity. Therefore, although L-type CCBs reduce calcium influx in vascular smooth muscle, thereby producing a vasodilatory effect, they do not block TRPV4-mediated Ca^2+^ signaling, which is critical for regulating the function of the endothelial barrier in the alveolar septa (Wu et al., 2009; Alkhedhairi and Aba Alkhayl, 2025). This mechanistic distinction explains why CCBs can cause pulmonary edema despite their vasodilatory effects: the edema does not result from the blockade of L-type channels in endothelial cells, but rather from the combined effects of increased hydrostatic pressure due to systemic vasodilation, uninhibited TRPV4-mediated Ca^2+^ signaling in endothelial cells, and impaired alveolar fluid clearance. Regarding the activation of inflammatory responses, CCB overdose may activate pro-inflammatory signaling pathways, leading to the release of pro-inflammatory cytokines such as tumor necrosis factor-α (TNF-α) and interleukin-1 (IL-1), triggering inflammatory reactions and exacerbating lung injury (Font et al., 2026; Leesar et al., 1994). These cytokines promote the recruitment and activation of neutrophils, enhance the expression of adhesion molecules on endothelial cells, and increase vascular permeability by disrupting endothelial cell junctions. The resulting inflammatory cascade exacerbates lung injury and may lead to the progression of NCPE to full-blown ARDS. Oxidative stress and calcium overload in alveolar epithelial cells may further exacerbate cellular damage and impair surfactant production and function. At the molecular level, the increase in cytokine production induced by L-type CCBs involves the activation of the transcription factor nuclear factor-κB (NF-κB). In vascular smooth muscle cells, CCBs such as nifedipine, verapamil, and diltiazem directly activate the NF-κB and NF-IL-6 pathways, leading to increased transcription of the IL-6 gene, a process that is independent of changes in intracellular calcium concentrations (Eickelberg et al., 1999). This calcium-independent mechanism is particularly important in ARDS, a condition in which NF-κB activation in pulmonary endothelial and epithelial cells drives the expression of various pro-inflammatory cytokines, including TNF-α, IL-1β, and IL-6, thereby exacerbating lung injury and promoting the progression from NCPE to ARDS. This pathway involves the phosphorylation and nuclear translocation of NF-κB p65, thereby enhancing the transcription of pro-inflammatory cytokine genes. In pulmonary microvessels, this CCB-induced NF-κB activation may act synergistically with the hemodynamic and permeability effects discussed above, creating a pro-inflammatory environment that exacerbates alveolar-capillary barrier dysfunction. These molecular-level insights provide a mechanistic link between L-type calcium channel blockade and the inflammatory amplification observed in CCB-associated pulmonary toxicity. Alveolar epithelial dysfunction is a characteristic pathological change in ARDS, including impaired pulmonary surfactant function and disruption of the alveolar-capillary barrier (Ware and Matthay, 2000); clinical observations following overdose of certain CCBs (such as verapamil) suggest a potential association with these processes. non-DHP CCBs have a more pronounced inhibitory effect on cardiac function, which may cause bradycardia and exacerbate heart failure, thereby indirectly leading to pulmonary fluid retention (Isbister et al., 2025). Iatrogenic factors, such as excessive fluid resuscitation to treat hypotension, may worsen pulmonary edema (Prigogine et al., 1991).

### 3.7 Clinical features and diagnosis

The clinical manifestations of CCB-related pulmonary toxicity primarily include acute dyspnea, cough, chest tightness, frothy sputum, and hypoxemia. In a small number of patients, symptoms may appear with a delay (e.g., NCPE occurring 3 days after amlodipine overdose) (Zambetti and Parrish, 2025). Key diagnostic criteria (St-Onge et al., 2017): 1. Confirm a history of CCB exposure (dose, duration, formulation, route of administration). 2. Clinical presentation consistent with pulmonary edema; echocardiography rules out left ventricular dysfunction (normal or slightly low pulmonary artery wedge pressure). 3. Chest X-ray or CT shows diffuse alveolar/interstitial infiltrates. 4. Rule out other causes such as heart failure, pneumonia, or pulmonary embolism.

Differential diagnosis is crucial for avoiding misdiagnosis. NCPE should be distinguished from cardiogenic pulmonary edema, which typically presents with elevated pulmonary artery wedge pressure (>18 mmHg), reduced ejection fraction, and jugular vein distension visible on echocardiography. In contrast, NCPE is characterized by normal or only mildly elevated wedge pressure and preserved cardiac function (Ware and Matthay, 2000). NCPE should be distinguished from pneumonia based on the presence of fever, productive cough, and elevated markers of infection (such as procalcitonin and C-reactive protein); the radiographic findings of pneumonia are typically lobar or segmental consolidation, rather than diffuse bilateral infiltrates. Aspiration pneumonitis typically occurs in patients with impaired consciousness or dysphagia, with infiltrates primarily located in dependent lung regions. Acute-onset dyspnea and pleuritic chest pain suggest pulmonary embolism, which can be ruled out by D-dimer testing and CT pulmonary angiography. In clinical practice, the definitive diagnosis of CCB-related NCPE requires consideration of the temporal relationship between drug exposure and symptom onset, exclusion of other causes, and resolution of symptoms following drug discontinuation and supportive therapy.

### 3.8 Treatment and prognosis

Treatment strategies should be tailored to the specific CCB subclass and type of shock (St-Onge et al., 2017; Isbister et al., 2025; Hong et al., 2022) (Table 1). The prognosis is generally favorable, and most cases recover completely following aggressive treatment (Simpson and Cole, 2024; Zambetti and Parrish, 2025); however, severe overdose can be fatal (Lota et al., 2008). Early recognition, prompt discontinuation of the drug, and precise hemodynamic support are key to improving the prognosis. A comprehensive algorithm for the management of CCB-associated pulmonary toxicity is provided in Figure 2.

**TABLE 1:** The treatment measures for CCB-associated pulmonary toxicity.

Treatment measures	Specific methods	Indications/Key notes
Detoxification	Activated charcoal, gastric lavage	Administer as early as possible to reduce absorption
Supportive Care	Oxygen therapy, fluid/electrolyte balance	Basic supportive measures
Mechanical Ventilation	Lung-protective strategies	Used for severe hypoxemia (ICU care)
Diuretics	Furosemide	To alleviate pulmonary edema; avoid hypovolemia
Vasopressors	Norepinephrine (Isbister et al., 2025)	For DHP overdose (vasodilatory shock)
Inotropic Agents	Epinephrine, high-dose insulin (Isbister et al., 2025)	For non-DHP overdose (cardiac depression)
Calcium Preparations	Intravenous calcium	Administer as early as possible; more effective for DHP
High-Dose Insulin	0.5–1 U/kg/h, up to a maximum of 10 U/kg/h (St-Onge et al., 2017)	For refractory shock; monitor blood glucose; be alert for delayed edema after discontinuation (Haq et al., 2025)
Extracorporeal Membrane Oxygenation (ECMO)	Venovenous or venoarterial mode	Used for refractory cases or massive overdose (Hong et al., 2022)
Fluid Management	Limit crystalloid infusion	To prevent worsening of pulmonary edema

**FIGURE 2:**
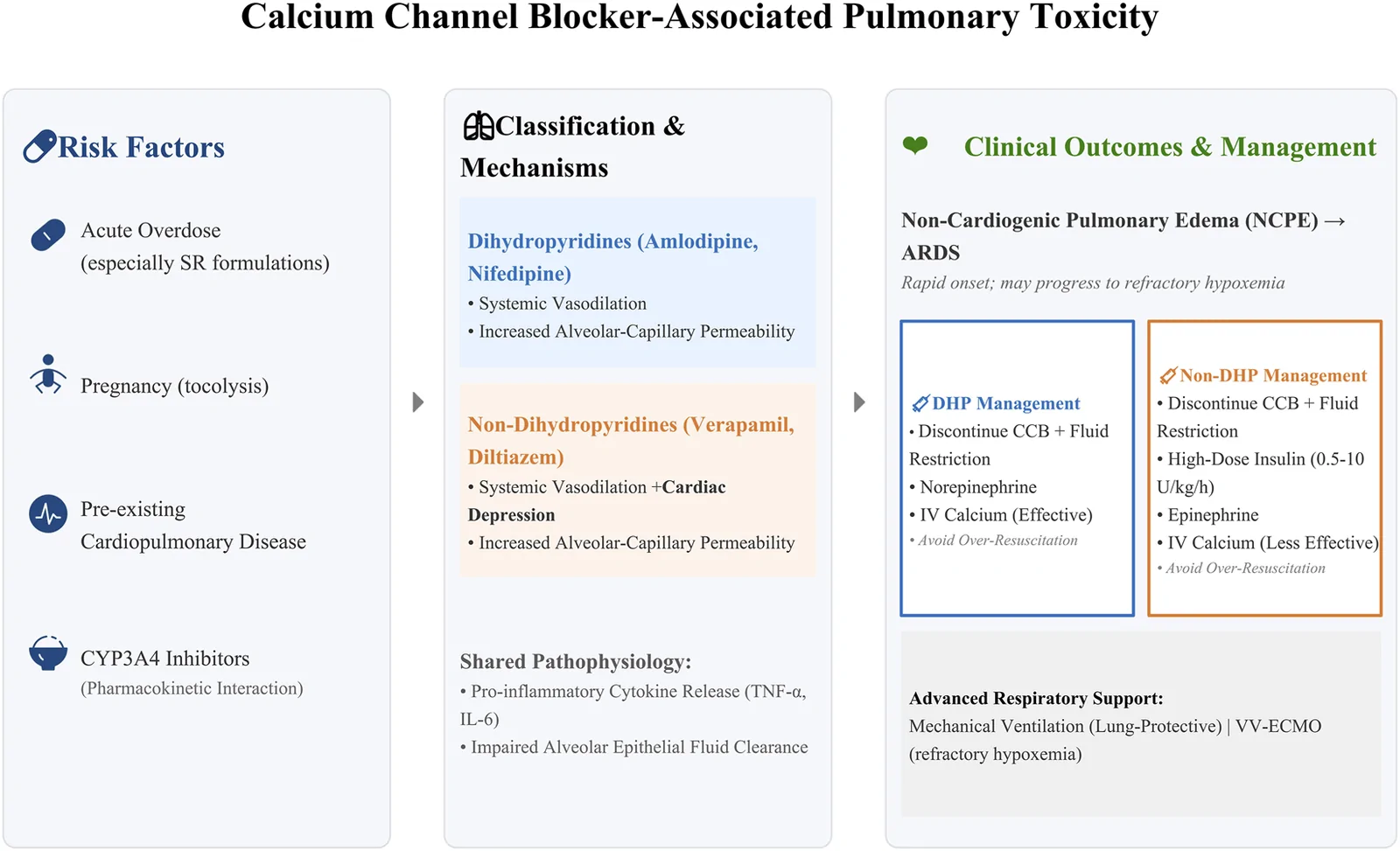
Calcium channel blocker-associated pulmonary toxicity.

## 4 Discussion

CCBs produce vasodilation, negative inotropy, negative chronotropy, and slowed AV conduction, positioning CCBs as first-line agents for hypertension, angina, and arrhythmias. However, the risk of pulmonary toxicity associated with CCBs must be adequately managed. This review systematically summarizes the current evidence regarding CCB-associated pulmonary toxicity. Key findings include: NCPE and ARDS are the two primary clinical manifestations; pharmacovigilance studies have identified significant signals linking nifedipine, diltiazem, and verapamil to pulmonary edema (Zhong et al., 2025; Ju et al., 2025); the reported proportion of NCPE reaches as high as 47% in cases of severe amlodipine toxicity (Lindeman et al., 2020); the reported occurrence of pulmonary edema is approximately 2.8% when nifedipine is used to inhibit uterine contractions (Spiesser-Robelet et al., 2015); and even low doses of amlodipine can induce severe NCPE, suggesting that individual susceptibility plays a significant role (Abboud et al., 2012). These key findings, together with the proposed management algorithm, are summarized in Figure 2.

A key distinction among CCB subclasses is that Isbister et al. clearly demonstrated that diltiazem and verapamil exhibit significantly higher toxicity than DHPs (Isbister et al., 2025). DHPs primarily cause distributive shock and capillary leakage through excessive vasodilation; non-DHPs, in contrast, involve a combination of cardiac depression and vasodilatory effects. This distinction directly guides treatment selection. Furthermore, the pathophysiology of ARDS involves disruption of the alveolar-capillary barrier and surfactant dysfunction (Ware and Matthay, 2000), providing a theoretical basis for understanding CCB-associated severe lung injury.

In special populations such as pregnant women, the use of nifedipine to inhibit uterine contractions significantly increases the risk of pulmonary edema; the signals reported in the FAERS system are extremely strong, so strict adherence to indications and enhanced monitoring are essential. It is important to note that pregnancy is a dynamic process involving fluid shifts, hormonal changes, and potential complications such as pre-eclampsia or peripartum cardiomyopathy. These factors can each independently lead to pulmonary edema and may complicate the determination of whether nifedipine is the sole causative factor. Therefore, when evaluating a pregnant woman who develops pulmonary edema following exposure to nifedipine, clinicians should systematically rule out other possible causes, including pre-eclampsia, peripartum cardiomyopathy, and fluid overload, before attributing the event to nifedipine as the sole precipitating factor. The diagnosis of pregnancy-related DIPE associated with nifedipine should be considered a diagnosis of exclusion. Although pediatric cases are rare, studies on the safety of long-term high-dose CCB use in children with pulmonary arterial hypertension (Wu et al., 2022), as well as case reports of ARDS resulting from amlodipine overdose in adolescents (Welsh et al., 2023), suggest that vigilance is equally warranted in the pediatric population.

First, clinicians should include NCPE in the differential diagnosis for patients presenting with acute dyspnea following CCB use. Second, monitoring and treatment strategies should be tailored according to the subclass of CCB (DHPs require close monitoring of blood pressure, while non-DHPs require concurrent monitoring of cardiac function and blood pressure) (Isbister et al., 2025; Hong et al., 2022). Third, monitoring of high-risk populations (pregnant women, patients with pulmonary arterial hypertension, and patients with hypertrophic obstructive cardiomyopathy) should be strengthened. Fourth, during management of overdose, excessive fluid resuscitation should be avoided, and vasopressors or inotropic agents should be selected based on the type of shock. Pharmacists, by actively monitoring, collecting and analyzing clinical medication data, can accurately identify signals of adverse drug reactions and provide early risk warnings for clinical practice (Li et al., 2026; Wang et al., 2023; Li et al., 2024). This is a crucial line of defense for ensuring the safety of patients’ medication use.

Several limitations should be acknowledged. First, the current evidence primarily relies on case reports, and large-scale prospective studies are still lacking. Second, the available incidence data are derived from only a few cohort studies, which limits the generalizability of our findings to broader populations or different clinical settings. Third, for certain CCBs, such as felodipine and nicardipine, data on pulmonary toxicity remain extremely scarce; however, this absence of evidence should not be interpreted as evidence of no risk.

Currently, there is a lack of prospective cohort studies or randomized controlled trials on CCB-associated pulmonary toxicity, which represents the most significant research gap in this field. Future efforts should focus on utilizing pharmacovigilance databases to quantify risks, conducting prospective cohort studies, exploring nitric oxide-mediated pathways and genetic susceptibility, and developing prevention and management guidelines tailored to different subclasses of CCBs and specific populations.

## 5 Conclusion

Although pulmonary toxicity associated with CCBs is rare, it can be severe, primarily manifesting as NCPE and ARDS. The risk is extremely low at standard therapeutic doses; however, heightened vigilance is warranted in cases of drug overdose, underlying cardiopulmonary disease, and pregnancy. The distinct toxicological profiles of different CCB subclasses dictate varying treatment strategies. Early recognition, prompt discontinuation of the medication, selection of vasoactive agents based on the type of shock, and aggressive life support are key to improving prognosis.
