# Supplementary material for: Calcium channel blocker-associated pulmonary toxicity: epidemiology, mechanisms, and clinical management
Source: Front Pharmacol. 2026 Sep 21;17:1892392. doi: 10.3389/fphar.2026.1892392 (PMC13635663; doi:10.3389/fphar.2026.1892392)
Supplement: Supplementary file 1 [file Table1.docx]

**Supplementary Table S1**. Complete Search Strategy in PubMed

| **Item** | **Details** |
| --- | --- |
| **Database** | PubMed |
| **Search Date** | May 2026 |
| **Search Query** | ("Calcium Channel Blockers"[MeSH] OR calcium channel blocker*[tiab] OR CCB[tiab] OR CCBs[tiab] OR nifedipine[tiab] OR amlodipine[tiab] OR verapamil[tiab] OR diltiazem[tiab]) AND ("Pulmonary Edema"[MeSH] OR "Respiratory Distress Syndrome"[MeSH] OR non-cardiogenic pulmonary edema[tiab] OR noncardiogenic pulmonary edema[tiab] OR ARDS[tiab] OR pulmonary toxicity[tiab]) AND 1980:2026[dp] AND (english[la] OR chinese[la]) AND humans[mesh] |
| **Limits Applied** | English or Chinese language; Humans; 1980–2026 |
| **Results** | 58 |
